# What About Fertility Staff Emotions? An Explorative Analysis of Healthcare Professionals’ Subjective Perspective

**DOI:** 10.5964/ejop.v16i4.2245

**Published:** 2020-11-27

**Authors:** Fabiola Fedele, Andrea Caputo, Barbara Cordella, Ludovico Muzii, Daniela Pietrangeli, Cesare Aragona, Viviana Langher

**Affiliations:** a Department of Dynamic and Clinical Psychology, “Sapienza” University of Rome, Rome, Italy; b Department of Gynecologic-Obstetrical and Urologic Sciences, “Sapienza” University of Rome, Umberto I Hospital, Rome, Italy; University of Palermo, Palermo, Italy

**Keywords:** infertility, assisted reproductive technology, healthcare professionals, staff’s narratives, emotional text analysis

## Abstract

Infertility-related psychological research is traditionally oriented to analyze the wellbeing of couples undergoing Assisted Reproductive Technologies (ART), than to study the job-related effects on the healthcare fertility staff. This piece of research aims at understanding the subjective perspective of the fertility professionals and contribute to identify their emotional dynamics in their work environment. An in-depth explorative research study was conducted on 12 healthcare professionals of an Italian ART hospital clinic. Structured interviews with open-ended questions were administered to explore their deep feelings about their professional experience. Emotional text analysis was then conducted to analyze the textual corpus of their narratives to grasp their affective symbolizations. Statistical multidimensional techniques were used to detect some thematic domains (cluster analysis) and latent factors organizing the contraposition between them (multiple correspondence analysis). Five thematic domains were detected which refer to different emotional dimensions, as follows: performance anxiety (Cluster 1), ambivalence between omnipotence and powerlessness (Cluster 2), care burden (Cluster 3), feeling of duty (Cluster 4), and sense of interdependence (Cluster 5). Then, four latent factors were identified dealing with the laborious attempt to remedy, the realistic sense of limitation, the incumbent feeling of pressure and the restorative sense of justice, respectively. The results are discussed based on the existing literature and some useful recommendations for staff education, training and clinical supervision are provided accordingly.

The problem of infertility and its consequent treatment (commonly known as Assisted Reproductive Technology [ART]) represents an increasing phenomenon, especially in industrialized countries. Infertility condition affects about 186 million people worldwide. On average, 9% of all couples in the reproductive age experience difficulties in conceiving spontaneously. In some regions of the world, infertility condition affect as much as 30% of couples (Stevenson & Hershberger, 2016). The term ART refers to medical treatments used to helpcouples conceive a pregnancy when it does not occur spontaneously, such as intrauterine insemination (IUI), in vitro fertilization (IVF), and third party-assisted ART (with gamete donors). Data from the Italian Assisted Reproduction Technology Register (IARTR) from 2005 to 2016 confirm the presence of constantly increasing percentages of children born after ART techniques in Italy (Scaravelli et al., 2016).

Several pieces of research have highlighted strong negative emotional reactions to procreative limitations such as frustration, pain, anger and dejection (Fassino, Pierò, Boggio, Piccioni, & Garzaro, 2002; Galhardo, Cunha, & Pinto-Gouveia, 2011; Vitale, La Rosa, Rapisarda, & Laganà, 2017; Wiweko, Anggraheni, Elvira, & Lubis, 2017). Infertility condition also appears to be related to feelings of powerlessness (Batool & de Visser, 2016; Benyamini, Gozlan, & Kokia, 2005; Cousineau & Domar, 2007; Cudmore, 2005; Nouman & Benyamini, 2019) and one’s own body (Clarke, Martin-Matthews, & Matthews, 2006). Indeed, diagnosis of infertility may trigger a self-deprecating sense of feminine or masculine personal deficiency due to failing “essentially to do what you're put on earth to do” (Schofield, 2015) and is perceived with shame, guilt, and reduced self-esteem (Yao, Chan, & Chan, 2018). Besides this, also the experience of infertility treatment represents as an overwhelming condition that dominates patients’ daily routine (Daniluk, 2001; Redshaw, Hockley, & Davidson, 2007) and may represent a critical moment because of the intrusion of medical procedures in the couple’s intimate life (e.g., injections, stimulations, oocyte pick-ups). In this regard, patients undergoing ART procedures may perceive treatment as a source of deep stress, anxiety and concern (Purewal, Chapman, & van den Akker, 2018; Stanhiser & Steiner, 2018), which increase with the progression of therapeutic failures, generating a sort of "failure syndrome" (Langher, Fedele, Caputo, Marchini, & Aragona, 2019; Milazzo, Mnatzaganian, Elshaug, Hemphill, & Hiller, 2016; Verhaak et al., 2005). Patients also show higher levels of depression and shame, compared to people with infertility problems who do not undergo ART (Galhardo, Pinto-Gouveia, Cunha, & Matos, 2011).

Despite previous research having deepened the patient’s psychosocial challenges of infertility, little is known about how healthcare members emotionally face infertility problems and treatment delivery. Only in recent times, an interest in examining stress, job satisfaction and emotional wellbeing of fertility clinic staff has been developed, given the role of stressors and perceived difficulties in effectively dealing with patients’ complaints and in achieving optimal pregnancy success rates (Gerson et al., 2004; Klitzman, 2018).

It is well acknowledged that patients with infertility problems challenge usual approaches to care, leading to potential difficulties in the therapeutic relationship (Gameiro, Boivin, & Domar, 2013; Grill, 2015; Leone et al., 2017; Schmidt, Tjørnhøj-Thomsen, Boivin, & Nyboe Andersen, 2005). From a psychoanalytic perspective (Fornari, 1976), medicine has generally two different functions respectively referring to curing disease as something separated from the patient (phallocentric function) and taking care of the good owned by the sick person (omphalocentric function). Instead, in assisted reproductive medicine, there is no disease to cure and treatment is not aimed at healing (Leone et al., 2017); therefore, the patient could be identified with his/her infertility condition that encompasses the entire self (Jaffe & Diamond, 2011). As well, the patient usually is not an individual but a couple that may have conflicting perspectives about treatment options and delivery (Leone et al., 2017). In this regard, triangular relationships may take place in the medical encounter, with the doctor representing a potential persecutor that is excluded or attacked by the couple or, vice versa, an ally in illicit coalitions with one of the two partners when the couple has opposite preferences (Shapiro, 2001). Besides, most of couples already know that there is a problem and develop excessive expectations about receiving an effective solution from clinicians (Fitzgerald, Legge, & Frank, 2013; Leone et al., 2017), posing unrealistic expectations and demanding an excessive amount of time and attention from medical staff (Boivin, Bunting, Koert, Ieng, & Verhaak, 2017; Grill, 2015). Therefore, due to the difference in expectations between medical staff and the couples, conflicting situations may emerge (Norré & Wischmann, 2011). In healthcare context, the concepts of transference and countertransference - as manifestations of unconscious mental activity - may help explain problematic care relationships (Goldberg, 2000; O’kelly, 1998); for instance, in the ART setting, medical staff may perceive a progressive defensive withdrawal and detachment towards patients as the result of the loss of control that patients project out (Castellano et al., 2011).

Besides, patients may be experienced as manipulative, entitled, dependent, self-destructive, non-compliant and hostile, thus raising negative feelings of frustration, anxiety, guilt and dislike among healthcare professionals (Grill, 2015). In this regard, such staff feelings may represent countertransference responses to what patients experience with regard to their infertility condition, as well as ambivalent emotions resulting from processes of idealization and devaluation enacted by patients (Castellano et al., 2011).

Patients undergoing ART are often dissatisfied with the level of empathy and attention received by the healthcare staff of the clinics where they are being treated (Gameiro, Boivin, & Domar, 2013; Grill, 2015; Schmidt et al., 2005). From such a perspective, negative patient-staff interactions can be stressful for the clinic staff (Fitzgerald, Legge, & Frank, 2013), leading to work burnout and lower job satisfaction. For example, in ART centers in the USA, about two third of medical staff agreed that the clinic environment was stressful (Gerson et al., 2004), especially because of time pressure (Simpson & Bor, 2001), work overload (Harris & Bond, 1987) and patients’ discontinuation (Boivin et al., 2012). In line with the broader literature on the doctor-patient relationship, which assumes an increase in medical stress as patients’ stress increases (An et al., 2009), patients’ psychological burden related to intrusive and demanding medical procedures may negatively affect the relationship with their doctors (Grill, 2015).

Besides, because of delivering bad news in the ART context, concerning infertility diagnosis and repeated treatment failures, healthcare staff tend to experience blame, fluctuating between the power of helping the couple generate life and the potential impotence emerging from treatment failure (Leone et al., 2017). This may increase the level of distress in fertility professionals, in turn negatively affecting their emotional reactions and overall quality of care (Boivin et al., 2012; Simpson & Bor, 2001).

Based on the review of the literature, the present manuscript aims at deepening this particular area of medical practice by exploring fertility staff emotions about their professional experience in the ART context. Emotions are not merely intended as primary response to the stimuli of the external reality, which pertain to the individual’s intrapsychic world (e.g., fear, rage, anxiety, joy, love, etc.). Rather, they mainly refer to the subjective experience resulting from affective symbolizations in terms of emotional meanings attributed to the reality (Carli & Paniccia, 2002). The sharing of affective symbolizations among individuals who coexist in the same context allows the establishment of implicit expectations regulating behaviors and consenting the development of interpersonal communication and adjustment (Petitta, Ghezzi, & Jiang, 2018). The inspection of shared affective symbolizations by ART professionals may provide some insights about the key aspects underlying their representations of ART work and care relationships.

In this regard, training and support programs for fertility clinical staff are advocated in order to reduce stress, prevent burnout and also improve the ability to care for patients (Gameiro, Boivin, & Domar, 2013; Grill, 2015; Norré & Wischmann, 2011; Patel, Sharma, & Kumar, 2018). As well, the need for providing staff training in communication-skills, shared decision-making, empathy, breaking bad news has raised in recent times, as well as the relevance of mental health practitioners in infertility clinics (Gameiro, Boivin, & Domar, 2013; Grill, 2015; Patel, Sharma, & Kumar, 2018). This may be particularly relevant in the Italian context, where the fertility staff’ experience is made more complex by ethical-religious issues and restrictive legislation, and third-party reproductive techniques, characterized by higher chance of success, have been allowed as a treatment option only in recent times.

To this purpose, we have decided not to refer to a priori and specific emotional indicators such as anxiety and depressive symptoms. Unlike the case of the aforementioned studies, we have formulated data-driven hypotheses by using professionals’ narratives. In fact, narrative represents a powerful strategy for making meaning of emotions and creating a sense of continuity about one's professional experience in facing work challenges. Besides, compared to self-reports methods, narrative ones have some advantages concerning the reduction of social desirability bias, the identification of specific nuances of an emotional experience, and the accuracy of data interpretations (Riessman, 2008).

Finally, the present manuscript deals with the emotional experiences of ART staff members in line with the previous research study focused on the experiences of patients with infertility problems (Langher et al., 2019). Both research projects were carried out in the same time period, in the same ART clinic and rely on emotional text analysis as used method. Despite this, they are independent research projects in terms of study aims, recruited samples, and analyzed interviews.

## Method

### Participants

Study participants included twelve ART professionals working for at least 12 months in the fertility clinic of a public healthcare hospital of Rome. Specifically, the purposive sample consisted of the director of the clinic, two gynecologists and two embryologists as permanent medical staff, two nurses and one midwife as permanent nursing staff, and four gynecology interns. Three were women and nine men; with half of the staff members having been working in the center for more than six years. Individual interviews were administered after having obtained informed written consent.

All the interviews were conducted and audio recorded inside the hospital, in a reserved and silent environment, to protect the privacy of the interviewees and keep personal information confidential. All the interviews were conducted when staff members were free from work activities, thus avoiding interference to their clinical practice. All data identifying participants were removed from the transcripts to guarantee anonymity.

The study received appropriate ethical approval from the Ethics Committee of the Umberto I Hospital in Rome.

### Materials

A structured interview was administered to the staff members of the fertility clinic to explore their deep feelings about their professional experience. The interview consisted of twelve open-ended questions, grouped into four broad categories referring to the professionals’ perception of: 1) one's professional function; 2) patients’ decision to undertake treatment; 3) treatment and its effects; 4) therapeutic relationship. The questions were created ad hoc based on a comprehensive search of the scientific literature conducted on the PsychINFO database on the topics of professionals’ perspectives in the context of infertility or fertility care, by using keywords such as: “work*”, “staff”, “team”, “health[care] professional* or provider*”, and “[in]fertility care or treatment”, “ART”, “IVF”, “IUI”. However, it should be acknowledged that the open-ended questions are just considered as prompts overall aimed at facilitating associative processes about the professional experience. Therefore, all the four broad categories previously mentioned make sense about the main research goal, allowing the exploration of the affective symbolizations pertaining the experience in the ART context, without representing well-established areas of investigation to be specifically addressed.

The staff members were encouraged to talk by following their free associations, offering them non-judgemental listening On average, the interviews lasted about 25 minutes, ranging from 15 to 35 minutes.

The interviews were administered from April to July 2017.

## Data Analysis

### Research Framework

Emotional text analysis (ETA; Carli, Paniccia, Giovagnoli, Carbone, & Bucci, 2016) was used to explore the study participants’ subjective experience, in line with previous research in healthcare settings (Caputo, 2014; Caputo, 2018a; Caputo, Giacchetta, & Langher, 2016) and with specific regard to fertility issues (Langher et al., 2019). From a methodological perspective, ETA is grounded on a psychoanalytic theory of language (Fornari, 1976) that, according to a ‘double reference’ principle, considers language as having both a lexical-cognitive (conscious) and symbolic-affective (unconscious) function. Whereas the lexical-cognitive function refers to rational and logic meanings conveyed by the source culture, the symbolic-affective one refers to subjective meanings based on shared affective sense-making processes. This is consistent with Matte Blanco’s (1981) bi-logic theory of mind, assuming that language can be intended as a rational instrument addressed to organize reality following an asymmetrical logic (i.e., intentional structuring or ordered constituent parts of language), but also the gateway to grasp the rules of unconscious processing following a symmetrical logic (i.e., syntagmatic relations between parts of language). According to the psychoanalytic technique of free association (Freud, 1966), such syntagmatic relations help researcher identify the emotional meaning of speech through deconstructing the typical linguistic links of the operational function of language to detect more spontaneous chains of associations between words (Carli & Paniccia, 2002). To this purpose, polysemy refers to emotional meanings attributable to a word, when it is extracted from the linguistic context, which allows the detection of two broad categories: Dense words (with high polysemy) featured by a high emotional value disregarding the specific linguistic context (i.e., “bomb” or “good”); and non-dense words ( characterized by low polysemy) that include empty words without significant content (e.g., articles, adverbs, conjunctions, auxiliary verbs) or ambiguous words with contradictory emotional configurations (i.e., “to presume” or “however”). The identified dense words, which are emotionally relevant and consistent with the research goal, can be grouped based on their co-occurrence, so to detect different symbolic domains (Caputo, 2013a).

### Textual Analysis Procedures

All the interviews were transcribed verbatim and combined in a single text corpus. Then, ETA was adopted through using the T-Lab (Version 9.1) software (specifically, “Thematic analysis of elementary contexts” procedure; Lancia, 2004), which performs both cluster analysis and multiple correspondence analysis from a digital “presence-absence” matrix with elementary context units (i.e., text segments) in rows and lexical units (i.e., words) in columns, respectively. Cluster analysis allows the groupings of text segments including the same co-occurring words with the highest probability (evaluated through the chi-square test), whereas multiple correspondence analysis allows the inspection of the relationship between such groupings in a multi-dimensional space (evaluated through the test value; Lancia, 2004). Groupings of words (clusters) represent symbolic domains and Cartesian axes (factors) are intended as latent dimensions organizing the contraposition between them. Clusters are labelled and interpreted by the researcher by using models of affective symbolization (Carli & Paniccia, 2002), which reveal different affective dynamics and interpersonal patterns, integrating constructivism and object relations theory (Caputo, 2013b; Caputo, 2018b; Langher, Brancadoro, D’Angeli, & Caputo, 2014) through a psychoanalytic method following an evidential and conjectural paradigm (Langher, Caputo, & Martino, 2017). In this regard, the focus of the textual analysis is on the identification of spontaneous chains of lexical associations, apart from their language context and the cognitive sense of the reported contents. Specifically, the interpretations about clusters and factors were independently formulated by three researchers and then jointly discussed, resolving potential discrepancies by consensus. Data interpretation is also based on the qualitative analysis of the interview extracts (i.e. text segments) as to get triangulation and semantic validity of data through cross verification. This was performed through comparing different sources (outputs of analysis about both grouping of words and the most characteristic elementary context units) and procedures (to infer the symbolic-affective meanings from the statistical word co-occurrence and to contextualize such meanings in terms of related contents from the participants’ direct quotations).

## Results

Overall, the text corpus includes 27,002 occurrences, thus falling in the 15,000-45,000 range characterizing small-medium sized texts (Bolasco, 1999). The percentage of distinct words (V = 3,510) out of the total occurrences (type/token ratio) is equal to 13%, whereas the percentage of hapaxes (H = 1,748) (i.e., words with only one occurrence) out of the distinct words is 49.8%. Therefore, a statistical analysis of the text corpus can be allowed, since type/token ratio and percentage of hapaxes are respectively lower than 20% and 50% (Bolasco, 1999). 

Table 1 shows the five groupings of text units and relative keywords emerging from cluster analysis, which are detected throughout the interviews and refer to different semantic domains shared by participants in terms of emotional symbolizations.

**Table 1:** Clusters With Most Characteristic Lemmas (Keywords) and Percentage of the Overall Elementary Context Units

Cluster 1 (16.00%) Performance anxiety		Cluster 2 (24.84%) Ambivalence between omnipotence and powerlessness		Cluster 3 (24.21%) Care burden		Cluster 4 (27.37%) The feeling of duty		Cluster 5 (7.58%) The sense of interdependence	
Lemma	χ^2^	Lemma	χ^2^	Lemma	χ^2^	Lemma	χ^2^	Lemma	χ^2^
Monitoring	85.07	Outcome	40.10	Need	58.52	Centre	21.30	Intern	61.55
Day	80.42	Satisfaction	22.92	Child	14.44	Number	17.46	Lab	50.51
Transfer	57.90	Great	19.19	Oocyte	12.98	Structure	14.05	Biologist	44.73
Pick up	52.44	Positive	12.27	Predisposition	12.62	Responsibility	14.05	Inside	30.92
Stimulation	47.01	High	12.20	To gather	12.09	Person	11.98	To occupy	28.39
Embryo	37.88	Frustration	10.96	Pain	11.57	User	10.70	Context	26.17
Follicle	37.30	Happy	10.90	Couple	11.52	To offer	10.70	Guard	26.17
Ultrasound	36.29	To achieve	9.07	Reproduction	11.51	Detachment	10.70	Porter	26.17
Procedure	31.13	Small	8.59	Stress	11.51	Commitment	9.34	To turn	26.17
To decide	20.62	To overcome	8.25	Complicated	8.63	Economic	9.34	Structured	20.42
Emotion	20.62	Failure	8.25	Spermatozoon	8.63	To Close	9.28	To devote	15.61
Negative	19.95	To search	8.16	Husband	7.81	Professional	7.85	Function	13.32
Therapy	15.95	To obtain	6.76	Male	7.15	Information	7.04	To change	12.62
Visit	15.40	Reality	6.69	Criticality	7.13	To work	6.02	Organization	12.62
To prescribe	15.40	Hope	6.02	Relationship	5.68	Normal	5.05	Service	7.58
Insemination	11.60	Pregnancy	5.46	Psychologist	5.67	Demand	5.05	Role	10.26
Anxiety	7.28	Result	5.43	Genetics	5.67	University	4.72	Gynecologist	8.68
Guide	5.23	Omnipotent	5.43	Partner	4.25	Public	4.60	Colleague	6.23
Expectation	5.23	To attempt	5.32	Delicate	4.19	To respect	4.18	To manage	5.72
Exam	3.96	Disappointment	3.97	To welcome	4.19	To succeed	3.99	To study	4.47

*Note*. The threshold value of Chi-square test (χ^2^) for each lemma is 3.84 (*df* = 1; *p* = .05). Textual data were translated into English only for the purposes of the manuscript.

## Thematic Domains

### Cluster 1: Performance Anxiety

This cluster includes 16.00% of the overall elementary context units. This thematic domain evokes a sense of worry and hypervigilance expressed through the control and constant effort about the use of ART procedures (“monitoring”, "ultrasound", “procedure”, “therapy”, “visit”), within a step-by-step path characterized by almost daily routines (“day”) and several medical performances ("transfer", "pick-up", “stimulation”, “insemination”). Indeed, these crucial ART-related actions are experienced by patients with high expectation because they allow fertility treatment continuation, thus evoking anxiety and fear for potential negative outcomes (“exciting”, “anxiety”, “expectation”, “exam”, “negative”). From this perspective, to handle such a performance anxiety, fertility specialists displace their attention from patient’s him/herself as a whole person to his/her fragmented bodily parts (“embryo”, “follicle”) as more controllable objects, on which they can exercise their expertise through embodying an assertive role ("to decide", "to prescribe”, “guide”).

“*In all weekly monitoring visits, patients always expect something more, one more follicle, and physicians to report something positive to them*” (Participant 2)

“*You have to be careful over the course of the entire path [...] this is a very difficult path, therapy is not easy, it is like an obstacle competition because everyday you have to pass an exam and you have high expectations*” (Participant 6)

### Cluster 2: Ambivalence Between Omnipotence and Powerlessness

This cluster includes 24.84% of elementary context units. This thematic domain describes both the fertility specialists’ sense of idealization and devaluation concerning their work. Therefore, it mostly focuses on ambivalent feelings of omnipotence and powerlessness, within the “attempt” to find a balance between an optimistic (“hope”) and realistic (“reality”) perspective about achieving the expected “outcome”. Each step of the therapeutic path entails, in fact, two extreme and antithetical alternatives, respectively resulting in a positive or negative outcome, without possibility of third options. Generating a new life, bypassing the obstacles of nature (“outcome”, “to achieve”, “to overcome”, “to search”, “to obtain”), represents a miraculous action that may activate emotions of satisfaction, greatness and power (“satisfaction”, “happy”, "great", "positive", "high", "omnipotence"). However, feelings of helplessness and debasement ("frustration", "small", "failure" and "disappointment") may emerge when clashing with the infertility-related insurmountable limits, making one's professional behavior useless and vain.

*“If you succeed to achieve the outcome your self-esteem increases* [...] *making a person happy is an important personal achievement even if you risk feeling omnipotent, but you have to remember that negative outcomes may break down your expectations”* (Participant 4)

“*Patients entrust the technological miracle. One may feel great expectation and also great frustration and grief when acknowledging to have failed, a possibility that is always around the corner, given the low success rates*” (Participant 7)

### Cluster 3: Care Burden

This cluster includes 24.21% of elementary context units. This thematic domain describes the staff’s attempts to deal with the procreative demand for ART, which is not limited to a biological reproductive function, but also involves the emotional sphere concerning the desire for a “child”. The burden of taking care of a “couple” as a unit rather than of single patients emerges, as highlighted by further keywords regarding the male “partner”, not just the woman’s body as object of medical attention ("husband" and "male"). This entails the fertility specialists’ difficulties when confronted with patients’ suffering ("pain", "stress", “need”). This occurs especially when providing partners with a diagnosis, shedding lights on the responsibility for infertility problems (“predisposition”, “oocyte”, "spermatozoon", “genetics”) or handling “complications” and “critical” issues related to treatment failure. The reference to “psychologist” may represent the search for relief from this emotional burden in the doctor-patient “relationship”, by relying on an alternative professional specifically charged with psychological and relational issues.

*“Couples are very delicate to deal with in the doctor-patient relationship* [...] *for me the most difficult moment is when the patient trusts you and begins to really get in touch with you”* (Participant 2)

“*What is mostly difficult is relating to the couple on a human level, especially when the outcome is not good* [...] *when you have to communicate bad news you feel troubled*” (Participant 8)

### Cluster 4: The Feeling of Duty

Cluster 4 consists of 27.37% of the elementary context units. This thematic domain mostly deals with the sense of professional responsibility, as highlighted by the reference to work effort in making the fertility service available to the entire population, trying to respond its procreative demand (“responsibility”, “commitment”, “professional”, “information”, “to work”, “to succeed”). Indeed, this cluster highlights that people undergoing ART procedures are not typical patients with a disease to cure, but persons who express a specific demand for realizing their procreative dream (“person”, “user”, “demand”). The high sense of responsibility is thus intertwined with the strong social relevance of infertility and its related intervention, intended as issues of public interest (“number”, "university", "public", “normal”). Therefore, the focus is on the duty of healthcare provision and its efficiency at the organizational management level ("centre", "structure", “to offer”), without judging the patient’s decision to resort on ART procedures, thus maintaining a neutral position (“detachment”).

“*This is a public center, so even those who do not have enough economic possibilities can access it, and at least try* […] *But we cannot judge, we must gather all those who come to the center”* (Participant 3)

*“You should get into the couple’s problem and try looking at it with detachment. I cannot do it because I feel very responsible about what I do. For example, if I have to choose a single spermatozoon and insert it (into the oocyte) I feel a very big duty”* (Participant 5)

### Cluster 5: The Sense of Interdependence

Cluster 5 consists of 7.58% of the overall elementary context units. It seems to evoke a sense of dependence that is expressed on several levels. First, the focus on the different professional “roles” and “functions” in terms of internal human resources of the fertility center ("resident", "biologist", “inside”, "guard", "porter", "structured", "gynecologist", “colleague”) highlights the relevance of teamwork and interdependence among professionals. Second, the constant need for adjustment and flexibility in terms of organizational management (“to shift”, “to change”, “service”, “to devote”, “to manage”) indicates the feeling of being subjected to a patient-centred service. Then, dependency-related feelings emerge also from the inevitable need for high qualification and further diligent fieldwork (“intern”, “lab”, “to study”) to effectively realize job goals. From such a perspective, interdependency among professionals may be perceived as a potential limitation within a context featured by unpredictability and risk of failure.

“*This is surely a teamwork, I mean that the gynecologist cannot perform his/her job without a well functioning lab and obviously the biologist in the lab cannot do his/hers without the gynecologist’s contribution*” (Participant 5)

“*The physician can be the best in the world but, if a biologist or paramedic does not work adequately, all teamwork gets destroyed. So all the staff members have to behave in a certain way because if someone makes a mistake, the outcome is devastating and this in turn affects users*” (Participant 7)

## Latent Factors

Four latent factors have been detected from correspondence analysis, which highlight the main semantic oppositions, based on the different positions of the identified clusters within the factorial space.

Table 2 shows the association between clusters and factors, expressed by relative contributions (squared cosines), indicating the quality of representation of each cluster on the different latent dimensions. The four latent factors explain the entire data variance (*R*^2^ = 100%). The sign reported in brackets (-/+) indicates the negative/positive factorial pole associated with each cluster.

**Table 2:** Associations Between Clusters and Factors (Relative Contributions)

Cluster	Factor 1	Factor 2	Factor 3	Factor 4
Cluster 1	**0.9047 (-)**	0.0379 (-)	0.0024 (-)	0.0549 (+)
Cluster 2	0.0350 (-)	**0.5004 (-)**	**0.1722 (+)**	**0.2924 (-)**
Cluster 3	0.0692 (+)	0.0569 (+)	**0.8352 (-)**	0.0387 (-)
Cluster 4	**0.5765 (+)**	0.0115 (+)	0.0850 (+)	**0.3270 (+)**
Cluster 5	0.0883 (-)	**0.7279 (+)**	0.1277 (+)	0.0560 (-)

*Note.* The sign reported in brackets (-/+) indicates the negative/positive factorial pole associated with each cluster. The highest associations between clusters and factors are indicated in bold.

**Figure 1:**
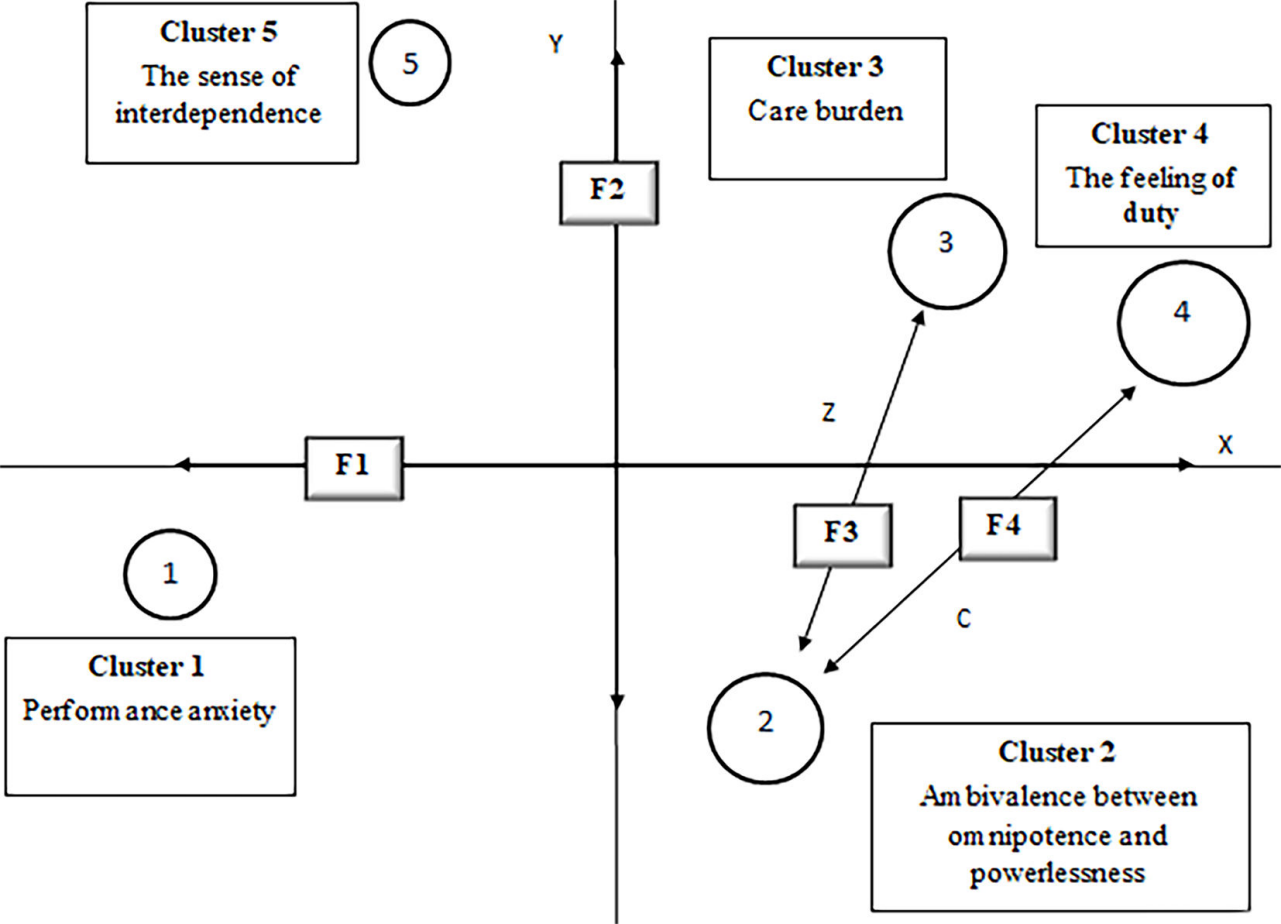
Factorial space.

Figure 1 represents the factorial space organizing the relationships between clusters and factors, thus providing a map of the emotional symbolizations shared by the professionals interviewed.

### The Laborious Attempt to Remedy (F1)

The first factor (39.96% of total variance) mainly differentiates Cluster 1 from Cluster 4. It refers to the laborious and solicitous attempt to remedy, at a symbolic level, infertility as damaged condition, accompanied by the fear for professional failure. On the one hand, it is expressed through constant and accurate attention to technical procedures to remedy patients’ deficient reproductive function (Cluster 1). On the other hand, it is expressed through the organizational effort to deliver a patient-centred treatment that is efficient and enables the realization of the couple’s procreative desire (Cluster 4).

### The Realistic Sense of Limitation (F2)

The second factor (26.44% of total variance) differentiates Cluster 2 from Cluster 5. This factor describes the sense of limitation in realizing one’s job. On one hand, it is expressed through feelings of powerlessness and uselessness when failing to overcome the obstacles of nature, given the poor success rates of fertility treatment (Cluster 2). On the other hand, it deals with the awareness of being dependent on other professionals, all having an essential role in ART context, thus relying on teamwork to effectively realize goals (Cluster 5).

### The Incumbent Feeling of Pressure (F3)

The third factor (20.22% of total variance) differentiates Cluster 2 from Cluster 3. It deals with the feeling of being under pressure in complying with patients’ expectations. On the one hand, it deals with the anxiety evoked by accomplishing positive outcomes that are highly invested on by patients but, at the same time, with low success rates (Cluster 2). On the other hand, it highlights the strong care burden in handling with the couple’s complexity, in terms of different underlying emotions and motivations to undergo ART (Cluster 3).

### The Restorative Sense of Justice (F4)

This factor (13.36% of total variance) mainly differentiates Cluster 2 from Cluster 4. It describes a restorative sense of justice, accompanied by the feeling of devoting oneself to a relevant mission. On one hand, it highlights the sense of fulfillment resulting from achieving ambitious outcomes through the ART “miraculous” capacity to restore the natural fertility of the couple (Cluster 2). On the other hand, it shows the sense of responsibility and duty in guaranteeing everybody’s access to fertility services, so to rehabilitate his/her image in the society (Cluster 4).

## Discussion

The results of this research study allowed the identification of the main emotional dimensions characterizing the experience of the staff members of an infertility center. In sum, the analysis identified five thematic domains (clusters) that describe the staff’s emotions emerging from their professional experience: performance anxiety, ambivalence between omnipotence and powerlessness, care burden, feeling of responsibility and sense of interdependence.

With regard to performance anxiety, it seems mainly related to the strive for achievement in performing a highly specialized, technical and demanding job (Fitzgerald, Legge, & Frank, 2013), as well as to the stressful conditions of the ART environment, involving work overload and time pressure (Gerson et al., 2004; Harris & Bond, 1987; Simpson & Bor, 2001). Consistently with previous research on ART patients (Langher et al., 2019), the ART treatment seems to be described as a step-by-step path consisting in huge obstacles to be overcome, which may induce anxiety feelings also in the fertility staff and predispose to an obsessional form of reparation (Boivin et al., 2012; Caputo, Fregonese, & Langher, 2018). Indeed, hypervigilance and constant effort to repetitive and structured technical actions could suggest a compulsive way to placate anxiety evoked by infertility as a damaged condition that one tries to remedy.

This great responsibility brings the healthcare staff to experience an ambivalent state fluctuating between the sense of power and of impotence, as found in other studies (Leone et al., 2017). Indeed, ART treatment is characterized by well-known low success rates, generally ranging from 4% to 40% (Centers for Disease Control and Prevention, 2016); accordingly, repeated failed treatment may explain for such a sense of powerlessness. As well, the miraculous possibility to generate life beyond nature can trigger feelings of grandiosity and over-estimation of one’s abilities (Castellano et al., 2011). This seems consistent with previous research highlighting that in caring work, helping professionals may resort to such a manic reparation, because they need to confirm that they have sufficient self-esteem to repair damage in others, so to defend themselves against failing to heal (Caputo, Fregonese, & Langher, 2018; Roberts, 1994).

Our results show fertility staff’s care burden as one of the major concerns, in line with a huge amount of literature (Fitzgerald, Legge, & Frank, 2013; Leone et al., 2017) reporting patients’ excessive expectations on clinicians, in terms of attention and effective solutions from medical staff (Boivin et al., 2017; Fitzgerald, Legge, & Frank, 2013; Grill, 2015; Leone et al., 2017), which negatively affects the therapeutic relationship (Gameiro, Boivin, & Domar, 2013; Grill, 2015; Leone et al., 2017; Schmidt et al., 2005). This seems specifically due to dealing with couples (not individuals) and complex demands for parenthood (not diseases), thus challenging some well-established healthcare assumptions about patient care (Leone et al., 2017).

The present study also found a feeling of duty of fertility staff that is intertwined with the strong social relevance of parenthood issues, which thus make ART treatment as a mean to rehabilitate the image of people with infertility problems in the community. In this regard, high social and family expectations on the value of procreation techniques exist, which may influence the personal decision to undergo ART (Cordella, Greco, Carlini, Greco, & Tambelli, 2018) so to pursue belonging and inclusion in the procreative world, as found in recent qualitative narrative research (Langher et al., 2019). Indeed, “voluntarily” childless is still portrayed as selfish and deviant in many socio-cultural contexts (Gillespie, 2001), especially for women, because motherhood is considered as a primary social role and infertility tends to be experienced as a “secret stigma” (Greil, 1991; Holmes, 2014).

Our findings show that teamwork and interdependence among professionals in fertility staff can represent a potential critical factor that may have a role in job satisfaction. Indeed, organization, team and management issues are often a potential source of work stressor, as highlighted by Boivin et al. (2017) who found that 60.4% of fertility health professionals reported team conflicts. Therefore, an interprofessional and multidisciplinary team approach in infertility treatment is considered as highly recommended, and was found to be effective for helping patients continue infertility treatment (Yano & Ohashi, 2010).

Based on the previously detected factors explaining for the subjective experience of the reproductive healthcare professionals involved in this study, we can synthesize the main emotional dimensions shaping their narratives, which can provide useful recommendations for staff education, training and clinical supervision.

The laborious attempt to remedy seems to feature the work of ART staff, that is constantly confronted with potential failure in realizing job goals despite the high technical and organizational effort. From such a perspective, ART professionals may feel an exaggerated and constant worry and get involved in compulsive activity, within the routine of repetitive and fragmented work procedures across the several steps of the ART treatment like in an assembly line. The central role of the technical nature of the job and of the high accuracy required may trigger depersonalization, contributing to shift the focus from the whole person to his/her bodily parts (e.g., uterus, eggs, embryos, sperm), thus looking at patients as childbearing machines (Gupta & Richters, 2008). The specific attention to the risk for depersonalization should thus be adequately included in staff training to prevent potential burnout. In this regard, humanization of care could be promoted in health professionals’ education, aimed at perceiving patients as complex and unique human beings who require acceptance, empathy, and active listening, through the valorization of meanings they assign to their demand for treatment.

The realistic sense of limitation emerges as a further key finding, concerning the actual power of action and effectiveness in delivering ART treatment. This can potentially lead to a diminished sense of personal accomplishment, mainly due to the low success rates and the needy dependency on other professionals to be able to achieve. Such conditions might make healthcare workers feel more uncertain about directly controlling the outcomes of one’s professional action and, in turn, engender unmotivation and sense of incompetence in helping patients. Therefore, it is important to improve the individual’s capabilities to mourn potential job failures, as well as to learn to look at teamwork as a resource rather than as a constraint, thus building trust and cohesion among colleagues through a multidisciplinary approach. In this regard, a multidisciplinary approach to treating infertility could be promoted through facilitating fertility team groups reflecting on the intervention effectiveness, not only in terms of offering the highest chances of pregnancy, but also of producing the best overall patient trust and satisfaction.

The incumbent feeling of pressure found in the present study seems to derive from anxiety in dealing with couples’ emotional complexity and expectations about the desire for a child. When failing to cure a disease, it is likely to rely on external attributions, such as task difficulty or bad luck in extirpating the evil. Instead, if the ART treatment fails to generate a new life, it is possible that this could be attributed to more internal factors, such as the lack of ability or effort of the staff in creating the good. This may lead to perceive patients as demanding, entitled and manipulative because they challenge the staff’s professional efficacy to a higher extent, in turn contributing to masked hostility and uncaring reactions. Clinical supervision could thus benefit from working on the ART professionals’ countertransference responses to patients, so to avoid potential passive-aggressive dynamics enacted in the therapeutic relationship. Therefore, joint discussion of clinical cases during supervision could be an opportunity to reflect on the critical incidents (e.g., conflicts, low adherence) in order to shed light on the working alliance with patients and the entire process of care. For example, informed consent could be used to improve patients’ awareness about their decision to turn to the ART treatment and to encourage a trusting relationship with the clinical staff.

Then, the restorative sense of justice in rehabilitating patients’ image in both biological and social terms through delivering fertility services represents the ART social mandate. This may engender a profound sense of responsibility to comply with social demands and expectations in ART professionals, beyond the mere sphere of medical intervention. Therefore, it could be useful to foster healthcare professionals’ awareness about the underlying values, ideals and ethical issues regarding the shared socio-cultural representations of fertility/infertility and parenthood. Indeed, the staff’s personal beliefs and implicit meanings regarding such issues may, to some extent, conflict with professional duties and the organizational mandate of fertility services.

Overall, the present study has some limitations, including the low generalizability of the findings due to the small sample size (*n* = 12) and the belonging of all the participants to the same ART clinic, thus making this piece of research comparable to a case study (Langher, Caputo, & Martino, 2017). Indeed, it should be noted that the entire ART staff population could be characterized by higher variability in the reported contents. Besides, given the exploratory aim of this qualitative inquiry, further quantitative studies are needed to confirm the interpretative frames here proposed. In this regard, future research could be conducted to develop new tools, based on the operationalization and measurement of the emotional dimensions emerging from the present study. In such a way, a wider sample could be included to test the robustness of the current findings in statistical terms. Besides, it could be interesting to deeply examine potential differences on the emotional dynamics characterizing the fertility staff, based on individual variables such as gender, professional role and years of tenure. As well, different groups of healthcare professionals (not working in the ART field) could be involved in comparison studies, to identify both some general aspects about the professional experience across diverse medical settings and the specificities referring to the ART context.

Despite these limitations, this study provides preliminary results allowing the better understanding of the subjective experience of the reproductive healthcare professionals, that is still scarcely examined in current literature, especially with regard to the Italian context. Besides, the use of narrative methods could be particularly useful in terms of action-research for staff education, training and clinical supervision to deliver more consistent, ecological and targeted interventions. The detection of the symbolic meanings referred to the professional function, patients, treatment and therapeutic relationship in the ART context may favor the professionals’ awareness about the implicitly shared beliefs, values and goals orienting their work practices. As well, this may help expressing and reflecting on potential ambivalent or conflicting aspects, thus making sense of one’s subjective experience. For example, representing the patient in terms of a fragmented body may be a strategy to remove the human component of care relationship, which is anxiety-inducing due to the patient’s high expectations. The sharing of such emotional issues among professionals may encourage the staff’s listening, mutual understanding and cohesion, with fruitful benefits in terms of teamwork and organizational climate.

In sum, the present manuscript provides interesting insights for developing the role of psychology into the medical management of the infertility. This is not only in terms of psychological consultations to couples undergoing ART, but also in terms of education, training and supervision to ART professionals with the aim of facilitating team work and care relationship.
